# Inequity in the use of physician services in Norway before and after introducing patient lists in primary care

**DOI:** 10.1186/1475-9276-10-25

**Published:** 2011-06-15

**Authors:** Astrid L Grasdal, Karin Monstad

**Affiliations:** 1 Department of economics, University of Bergen, Pb 7802, 5020 Bergen, Norway

## Abstract

**Background:**

Inequity in use of physician services has been detected even within health care systems with universal coverage of the population through public insurance schemes. In this study we analyse and compare inequity in use of physician visits (GP and specialists) in Norway based on data from the Surveys of Living Conditions for the years 2000, 2002 and 2005. A patient list system was introduced for GPs in 2001 to improve GP accessibility, strengthen the stability of the patient-doctor relationship and ensure equity in the use of health care services for the entire population.

**Method:**

We measure horizontal inequity by concentration indices and investigate changes in inequity over time when decomposing the concentration indices into the contribution of its determinants.

**Results:**

We find that pro-rich inequity in the probability of seeing a private outpatient specialist has declined, but still existed in 2005.

**Conclusion:**

Improved patient-doctor stability as well as better GP accessibility facilitated by the introduction of patient lists improved access to private specialist services. In particular the less well off benefited from this reform.

## Background

Countries that pursue equity in health among their populations increasingly acknowledge equity in health care utilization as an important intermediate aim to approach this goal. Empirical analyses generally show that these countries tend to succeed well at the primary health care level [1,2]. For the utilization of specialist medical care, the picture is different. Inequity in favour of the well off tends to be the rule rather than the exception. Even in countries with universal coverage of the population through public insurance schemes that ensure access to high quality services at low or no financial cost, richer and better educated tend to use more specialist care, conditional on need and other non-need factors [3].

Research on implications of the organization and finance of health care for equity in utilization is limited. Some studies provide evidence that growing markets for private supplementary health insurance promote inequity in physician visits [4-6] In two cross-country comparisons of inequity in physician visits for the EU [2] and the OECD [1] one seek to explain variation in inequity of utilization by differences in features of health care systems. Copayment, permission for specialists to work in dual (public/private) practices, and high degree of private provision of insurance and/or specialized medical care are all features associated with a higher degree of inequity in the use of medical specialists. While the gate keeper role of general practitioners (GPs) seems to mitigate inequity in GP visits, gate keeping is also associated with larger education gradients in the utilization of specialized medical care [1].

In the current analysis we seek to shed additional light on the role institutional features of GP practice may have for equity in doctor utilization in Norway. In June 2001 the Regular General Practitioner (RGP) Scheme was introduced, and a patient list system was established for the entire population. The main objectives of the reform were to improve access to GP-services and facilitate more stable patient-GP relationships [7]. We investigate if the reform had unintended implications for equity in GP- and medical specialist utilization by comparing the distribution of utilization by income before and after the reform, using three years of comparable cross-sectional data from the Norwegian Survey of Living Conditions (SLC). We do not claim that causal effect on equity of the reform is identified in this analysis. Nevertheless, from a health policy perspective, it is of interest to establish whether equity actually has improved or been harmed after such a large change in the primary health care system, in particular since provision of services according to need, irrespective of place of residence, socioeconomic conditions or ethnic background is stated by law as principle for provision of health care services [4,5].

We find that income-related inequity in the use of private specialists has declined but still existed in 2005. Our analysis shows that the decline is linked to changes in the responsiveness of utilization to individual socio-economic characteristics, and we argue that it is reasonable to link the observed changes in behaviour with respect to health care utilization to the introduction of the list patient system.

In what follows, we first describe the Norwegian system for provision of physician services. In section 3 we discuss expected effects of the GP reform. Section 4 presents the method and section 5 the data. The analysis and the results are presented in section 6 while discussion and conclusions are given in section 7.

## 2. Provision of physician services in Norway

The Norwegian health care system is characterized by tax-financed public provision and universal coverage [9]. Priority assignment of patients is regulated by the Act on Patients Rights and the Act on Health Enterprises with the aim to provide high quality care dependent upon need, and independent upon socioeconomic background characteristics and place of living. Below we explain how the provision of health care services is divided between the levels of government, and comment on changes that took place during the years 2000-2005.

### Primary care

Primary health care is the responsibility of the municipalities. Most GPs are self-employed with contracts administrated by the municipalities. By the end of the 1990s, many municipalities, in particular in rural and remote regions, experienced difficulties attracting new GPs for vacant positions. Temporary contracts and a general shortage of GPs resulted in frequent shifts of GPs and long waiting times for regular consultations. The RGP Scheme was introduced in June 2001 to attract more GPs, to facilitate stability in the patient-doctor relationship and improve equity in physician utilization. With the list patient system, GPs were responsible for the provision of primary health care services to patients belonging to his or her list. At its introduction, all inhabitants were presented with a list of available GPs and asked to select three preferred GPs. Seventy-four per cent sent in this form, and ninety-two per cent of them were listed with one of their preferred choices [10]. The rest were given notice that they had been tentatively assigned to a GP. In most cases, this assignment was accepted, but inhabitants also had the right to change to another GP, a right that is a permanent feature of the list patient system. GPs were not allowed to refuse access to their list as long as they had vacant slots on their lists.

The RGP Scheme also changed GPs' remuneration criteria, but a mixed system was kept throughout. A major component is fee-for-service, where fees are paid partly by patients themselves and partly by the National Insurance Scheme. Before the RGP Scheme was introduced, GPs received a practice allowance from the municipality, the size depending on the number of auxiliaries. After the reform, practice allowances were replaced by a capitation component depending on list size. Approximately 30% of the income earned by GPs is expected to come from capitation and the rest from fee-for-service. The former component used to represent a larger share of the GP's total income before the reform. Exceptions to the mixed system are found in some of the small and thinly settled municipalities where GPs have been given a fixed salary both prior to and after the GP reform.

Both prior to and after the reform, GPs served as gatekeepers for medical treatment by a specialist and for elective hospital treatment but this system was not fully consistent, as direct access for some specialised services existed. Parallel with the reform the gatekeeper system became more restrictive as referral was made mandatory in order for the specialist to claim reimbursement from the National Insurance Scheme. Without referral the patient can still consult the specialist directly but will then have to pay an extra copayment. Consultation with private GPs operating outside the patient list system has also been possible both before and after the reform. These GPs are easier to access in urban areas, but must be paid fully by the patients themselves.

Nearly all GPs signed up for the RGP scheme, and it also attracted new GP entrants. According to municipality statistics the number of physician-labour years per thousand inhabitants in primary care increased on average from 1.06 in 2000 to 1.15 in 2005. The increase was larger in large municipalities and in municipalities with growing populations. Already when the reform became effective in 2001, 94,6% of the population signed up on a list with a GP. By 2005, 98.5% of the population had signed up. Of these, only 1.3% were signed up on lists still without a regular GP.

### Specialized outpatient medical care

Out-patient specialist health care services are provided by public hospitals and by private, self-employed medical specialists or clinics serving the state owned regional health enterprises (RHE) (owned by the counties before 2002). Provision and funding of services are based on contracted agreements. There is some scope for competition in elective treatment as patients are free to choose their provider on a national basis, but few patients receive treatment outside of the hospitals' natural catchment areas [11]. Private providers are paid on a fee-for-service basis. Medical specialists employed by public hospitals are salaried. They were also allowed to work extra hours in private practices outside the hospital (dual practice). For patients, the level of copayment is the same regardless of whether treatment is received from a public or private provider as long as the private provider has a contract with an RHE and the patient is referred by a GP.

From 2000 to 2005, the number of private specialists on a contracted agreements with regional health enterprises was stable, but their activity level grew (yearly growth was 8.9%, 10.3% and 4.3% for the years 2002-2005 [12]. For specialised outpatient treatment in hospitals the growth in activity level was more moderate (5.9%, 4.1% and 2.5% for the same period).

As referred to in the introduction, several studies have pointed to private health insurance as an important contributor to inequities in health care utilization in specialist care. In Norway, private health insurance was virtually non-existent in 2000. Since then, a growing number of private insurance companies offer supplementary private health insurance that guarantees specialist examination and treatment shortly after referral from a GP. By 2005, about 60,000 persons, corresponding to approximately 2.3% of the labour force, had private supplementary health insurance [13].

## 3. Expected effects of GP reform

The introduction of the RGP Scheme changed GPs' financial incentives as well as the character of the patient-doctor relationship. In the following, we will discuss how these changes might affect income-related inequality in use of services.

The introduction of capitation payment could induce GPs to increase the referral rate to provide time for new patients [14]. The impact of the RGP Scheme on referrals is particularly interesting from an equity perspective, given that GPs are prescribed a gatekeeper role and that specialist services was found to be inequitably distributed before the reform [1]. The introduction of a list patient system gave a larger part of the population access to a stable patient-doctor relationship and should imply that the GP knows the patient better, so that there is less need to refer to specialist. This was the effect that policy-makers expected [15]. On the other hand, contracting the patient -doctor relationship also increased responsibility for the patients, which could cause a tendency for the GP to refer, "just to be sure." The introduction of capitation also provides the GPs with an incentive to compete for patients by offering high service quality [16], and patients may perceive having a referral as good quality. In a qualitative study, GPs themselves express that they perceive their gatekeeping role as weakened after the reform [17]. The only quantitative study on how the RGP Scheme has impacted on referrals indicates a rise in referral rates [18]. Their analysis focuses on whether the patient had a regular GP or not, and not on the relationship between referrals and patient SES.

While we find it reasonable to expect more referrals because of the stronger advocate role and the effect of competition, the distribution according to SES is not obvious. On the one hand, several factors could lead to more inequity in favour of high SES patients: these patients may be more attractive customers to a GP who wants to have a large list of patients, because their average health is better, as shown for instance in [19]. They may also be more demanding, aware of their rights within the health care system, or they may be better informed and have better communicative skills and therefore be more able to present the severity of their illness [20] On the other hand, the reform could have a greater impact on low income patients because they are overrepresented in remote areas, where discontinuity in the doctor-patient relationship was particularly frequent before the reform. Having access to a GP who had a particular responsibility for them could also give these patients better access to specialist services. Furthermore, the more restrictive gate-keeping regulation for referrals affected patients who before the reform went straight to the specialist, and high SES individuals are overrepresented within this group. After the reform, some of them may have decided to drop the consultation altogether or chosen to be treated by the GP instead. In sum, it is unclear whether we should expect the RGP Scheme to increase or decrease inequity in use of specialist services, and we leave this question for empirical investigation.

## 4. Methods for measuring and explaining inequity

We are interested in how the use of health services, denoted *y*, is distributed according to income when *y *represents six different outcomes: the number of visits and the dichotomous, for GP, private specialist, and hospital outpatient visits, respectively. Our survey data does not allow us to measure the number of private specialist visits that are financed privately, i.e., out-of-pocket or by private insurer, as opposed to publicly financed. We know of no other source of data that could inform us on this [21].

There is a clear distinction between inequality and inequity. While inequality simply refers to whether there is a correlation between a person's use of services and the person's ranking in the income distribution, inequity on the other hand takes into account individual need for treatment. Both inequality and inequity can be measured by concentration indices, which facilitate a comparison across different types of health care services and over time. The methods used are thoroughly described in [22]. Income-related *inequality *in use can be expressed by means of a concentration index *CI*, which is a measure of relative inequality:(1)

where *μ *is the mean of *y *and R*_i _*is the fractional rank of the *i*th person in the income distribution. The index ranges between (-1) and (+1) and takes a positive (negative) value if the health care variable is concentrated among the rich (poor).

To study the development of *inequity*, we first compute a concentration index for horizontal inequity, *HI*. To account for need, *HI *is estimated based on a regression of *y *on explanatory variables *x*. The dependent variables studied call for non-linear models, and we have used a probit model for the dichotomous outcomes variable and negative binominal models for the number of visits. Given a defined set of need-variables *x^N ^*and non-need variables *x^NN^*, the need-expected level of care can be predicted for each individual, which for a non-linear model is contingent upon the level of *x^NN^*. Note that the non-need variables include income. Setting non-need variables equal to their sample means, need-predicted health care use  is estimated as [23]:(2)

HI is a concentration index for estimated individual need-standardized use , which is found as:(3)

Computing an HI index as explained above requires making explicit value judgements about what should be defined as "need" and "non-need" variables. Our second approach to analysing inequity leaves this classification task to the reader, as inequality is decomposed and attributed to the covariates *k *as proposed by [24]. Note that the set of covariates included will not influence the inequality index (CI), but potentially the horizontal inequity index (HI). To apply the decomposition method to non-linear explanatory models, we use a linear approximation [25]:(4)

where  are partial effects of each variable treated as fixed parameters and evaluated at sample means, *C_k _*is the concentration index for variable *k *and *GC*_*ε *_is the generalized concentration index for the error term and can be computed as a residual. The product  is covariate *k*'s contribution to the total inequality observed.

Our main interest is in the *change *in inequity over time, which can be analysed using an Oaxaca decomposition [24,26]:(5)

or, using alternative base years:(6)

Thus, there are two sources for changes in the concentration index: First, over time, the underlying determinants *k *may become less or more concentrated with respect to income. Second, health care utilization may become more or less responsive to the *k *variable. The latter source is captured by changes in η _k_, which is called the elasticity of variable *k*.

It should be stressed that the analysis is descriptive and does not claim to reveal causal relationships.

## 5. Data

We use data from two different sources of SLC. Statistics Norway (SN) conducts annual theme-rotating, cross-sectional surveys. Every year 5000 persons aged 16+ are drawn according to SN's general sampling plan (institutionalized persons are excluded). Data regarding working and living conditions and health are collected through a combination of personal interviews and postal questionnaires. In addition, data are merged with administrative records with information regarding income, social insurance benefits and education. SN also conducted a panel survey covering the years 1997 to 2002. For this survey, a separate sample of 5000 representative individuals aged 16+ was drawn, with additional 16-year-olds added every year. All individuals included in this sample are approached every year regardless of former response behaviour. Collection of data is conducted according to the same procedures as in the cross sections, and formulation and selection of questions in the panel and the cross sections are to some extent overlapping across years. Since the questions regarding doctor utilization and several other relevant questions are identical in the 2000 wave of the panel and the cross sections in 2002 and 2005, these years' samples were the natural candidates for our analysis.

In all three years, respondents were asked about GP and medical specialist visits. For GP visits, the questions used are twofold: i) Have you, during the past 12 months, because of your own health condition, consulted a GP? And, if so, ii) how many times during the last 12 months have you consulted a GP? For medical specialists, there are separate questions for out-patient consultations in hospital and consultations with private specialist/clinic, and for both cases it is asked for any visit and, in addition, in the case of at least one visit, for the total number of visits. Based on this, our chosen outcome variables are 1) the dichotomous for having any visit or not, and 2) the continuous for number of visits to a GP, a private specialist, or a hospital outpatient clinic, respectively.

For the need-standardization of utilization, we include in addition to age-gender dummies, the standard measure of health based on responses to the question on self-assessed health status as either very good, good, neither good nor bad, poor or very poor. In 2002 and 2005, we have more data on health status. In these years, we can also include two questions regarding presence of any chronic physical or mental health condition and the possible degree of limitation in daily activities because of this, as well as a variable counting the number of conditions reported when respondents are presented with a list of 50 different diseases and health problems. In all regressions, we follow [1] and include variables not directly related to need or health status but still relevant for the utilization of health care services. Educational level, marital status and country of origin are expected to affect the efficiency of health production and the propensity to seek care, while activity status and region of residence are expected to affect the time price of health care use. In the Norwegian setting, region of residence is also expected to capture differences in access to medical services, as many medical specialist services are located in urban areas, and in the capital and surrounding areas in particular.

The response rate in the SLCs is stable at around 70%, with the main reason for non-response being refusal to participate. We decided to focus on the age group 16-69, because there are few individuals aged 70-79 in the 2000 sample, and we wanted to have the samples comparable with respect to age composition as population aging in itself leads to increasing income related inequality in health over time [27]. After excluding observations with missing data on one or more variables except educational level, we are left with 3371 observations in 2000, 2965 in 2002 and 3002 in 2005. As immigrants typically are over-represented among respondents with missing data on education, we decided to keep these observations in the sample and control for this in the regression analyses by including a dummy for missing data on education. Means and standard deviations for dependent variables and covariates in all three samples/years are listed in Table 1. For utilization of physician services, we see that the percent of the respondents having consulted a GP at least once during the last 12 months is somewhat lower (70%) in 2005 than in 2000 (72%). The average number of GP consultations as well is lower in 2005. The probability of seeing an outpatient medical specialist is 35-38%. We see a shift from using private options to public specialists from 2000 to 2005 and for two of the outcome variables the changes in means are statistically significant.

**Table 1:** Means of dependent variables and covariates (std.dev. in parenthesis)

	2000 (n = 3371)		2002 (n = 2965)		2005 (n = 3002)	
*dependent variables:*						
probability of GP visit ^a)^	0.723		0.728		0.697	
number of GP visits	2.934	(4.355)	2.798	(4.198)	2.771	(4.548)
probability of private specialist visit	0.164		0.171		0.157	
number of private specialist visits ^a)^	0.387	(1.708)	0.343	(1.489)	0.305	(1.211)
probability of hospital outpatient visit^a)^	0.184		0.217		0.208	
number of hospital outpatient visits	0.425	(1.815)	0.478	(1.483)	0.448	(1.369)
*explanatory variables:*						
log of household taxed income ^c)^	12.199	(0.537)	12.302	(0.524)	12.347	(0.654)
self-assessed health very good ^b)^	0.295		0.355		0.397	
self-assessed health good	0.508		0.483		0.441	
self-assessed health fair	0.142		0.113		0.108	
self-assessed health poor	0.047		0.045		0.042	
self-assessed health very poor	0.008		0.005		0.011	
number of specific diagnosis			0.487	(1.026)	0.469	(0.973)
no chronic disease ^b)^			0.642		0.659	
chronic disease, no limitations			0.045		0.041	
chronic disease, some limitations			0.231		0.212	
chronic disease, severe limitations			0.082		0.088	
male, age 16-29 ^b)^	0.133		0.123		0.130	
male, age 30-44	0.161		0.164		0.161	
male, age 45-59	0.137		0.162		0.149	
male, age 60-69	0.055		0.062		0.067	
female, age 16-29	0.125		0.124		0.124	
female, age 30-44	0.175		0.160		0.157	
female, age 45-59	0.152		0.140		0.142	
female, age 60-69	0.061		0.066		0.069	
completed compulsory schooling only ^b)^	0.144		0.124		0.116	
completed <3 years upper secondary	0.325		0.290		0.272	
completed 3 years upper secondary	0.265		0.297		0.298	
completed > 3 years upper secondary	0.265		0.274		0.270	
missing data on education	0.005		0.015		0.044	
region 1 (capital and surroundings) ^b)^	0.219		0.210		0.221	
region 2(eastern except capital area)	0.263		0.266		0.277	
region 3 (south-west)	0.142		0.149		0.129	
region 4 (west)	0.175		0.167		0.181	
region 5 (middle)	0.097		0.103		0.089	
region 6 (north)	0.104		0.106		0.103	
population>20000 individuals ^b)^	0.432		0.432		0.457	
population 2000-20000 individuals	0.255		0.254		0.253	
population <2000 individuals	0.314		0.314		0.290	
single ^b)^	0.245		0.227		0.260	
married	0.521		0.517		0.464	
cohabitating	0.144		0.172		0.183	
divorced	0.067		0.066		0.070	
widow/widower	0.023		0.017		0.023	
working > = 30 hours a week ^b)^	0.606		0.602		0.567	
disabled	0.070		0.085		0.088	
student	0.064		0.064		0.088	
doing military service	0.004		0.004		0.002	
working part time	0.153		0.149		0.145	
inactive in labour market	0.103		0.096		0.110	
born in Norway ^b)^	0.943		0.935		0.916	
born in Europe except Norway	0.033		0.036		0.048	
born outside of Europe	0.024		0.029		0.036	

a) When comparing means and proportions in 2000 to 2005 figures, two-tailed test shows that the difference is statistically different from zero at the 5% level. The test is undertaken for all dependent variables.

^b) ^Reference category.

c) Income is NOK 2005, OECD scale equivalised.

## 6. Results

Our examination of inequity takes as its starting point the observed, unstandardized income-related inequality in health care utilization. In all years, inequality indices (not reported here) concerning GP care and hospital outpatient use are negative, reflecting that use is concentrated among the poorer income groups. In contrast, the distribution of private specialist services is pro-rich, except for number of visits in 2005.

Our main results are based on equations (1)-(3) and reported in table 2. The horizontal inequity indices for the main specification are shown in the three columns to the left of the table, where "need" is defined by age, gender and self-assessed health, while the two last columns utilize more health information available for 2002 and 2005 only: the number of specific diagnosis reported and the existence and severity of a chronic disease. For most health delivery outcomes, the estimated inequity index is not statistically significant. However, for the probability of a private specialist visit the index is statistically significant. Use is distributed in favour of the rich both in 2000 and 2002 in the main specification. The estimated inequity decreases considerably over the five-year period. Still, we see that for all health utilization measures, additional data on health make the indices turn more pro-rich (or less pro-poor). Inequity in the use of a private specialist persists in 2005, with an inequity index of 0.0472. Therefore our further analysis focuses on inequity in the probability of a private specialist visit. As the definition of inequity is a controversial issue, we will make our analysis of inequity more transparent by a decomposition analysis of inequality. We focus on 2000 and 2005, and on the change in CI over time. First, we examine which factors contribute the most to inequality by decomposing the CI indices year-by-year; see equation (4). Overall, marginal effects and single variables' elasticities have the expected sign; see table 3. Although the propensity to visit a private specialist is higher the poorer is self-reported health (see Table 4), the elasticity decreases with ill health. This is the result of lower means: for instance, in 2000, the proportion reporting "good health" was 50.8% while the proportion reporting poor or very poor health was only 5.5%.

**Table 2:** Inequity in the use of doctor services - horizontal inequity indices

	2000	2002	2005	2002	2005
**Dependent variable **^**a)**^	(I)	(I)	(I)	(II)	(II)
probability of GP visit	0.0091	-0.0031	0.0050	0.0013	0.0068
	*1.49*	*-0.49*	*0.74*	*0.20*	*1.03*
number of GP visits	-0.0109	0.0053	-0.0066	0.0207	0.0072
	*-0.78*	*0.37*	*-0.38*	*1.48*	*0.41*
probability of private specialist visit	**0.0726**	**0.0496**	0.0449	**0.0571**	**0.0472**
	*3.31*	*2.16*	*1.87*	*2.50*	*1.97*
number of private specialist visits	0.0612	0.0622	0.0311	0.0781	0.0338
	*1.23*	*1.59*	*0.72*	*1.94*	*0.77*
probability of outpatient hospital visit	0.0275	0.0143	-0.0043	0.0222	0.0036
	*1.37*	*0.74*	*-0.22*	*1.16*	*0.19*
number of outpatient hospital visits	0.0381	-0.0021	0.0244	0.0129	0.0415
	*0.81*	*-0.07*	*0.79*	*0.43*	*1.36*

a) Specification (I) uses self-assessed health as the only health need indicator, while (II) includes additional health information. t-values are given in italics below each index. Statistically significant indices at 5% level are shown in bold. Horizontal inequity indices (HI) are based on equations (1)-(3). To compute HI, we apply eq. (1) but replace y_i _in (1) with  from eq. (3).

**Table 3:** Inequality decomposition for probability of private specialist visit, 2000 and 2005

	Elasticities ^a)^		Concentration indices		Contributions ^b)^	
	2000	2005	2000	2005	2000	2005
self-assessed health good	0.129	0.187	0.011	-0.020	0.0015	-0.0037
self-assessed health fair	0.091	0.128	-0.047	-0.060	-0.0043	-0.0077
self-assessed health poor or very poor	0.084	0.087	-0.206	-0.232	**-0.0174**	**-0.0201**
male, age 30-44	-0.017	-0.006	0.028	0.024	-0.0005	-0.0001
male, age 45-59	0.033	-0.006	0.243	0.201	0.0080	-0.0012
male, age 60-69	0.041	0.014	0.010	0.138	0.0004	0.0020
female, age 16-29	0.067	0.012	-0.175	-0.265	**-0.0117**	-0.0031
female, age 30-44	0.101	0.032	-0.054	-0.043	**-0.0055**	-0.0014
female, age 45-59	0.085	0.093	0.198	0.182	**0.0168**	**0.0169**
female, age 60-69	0.026	0.043	-0.250	0.013	-0.0064	0.0006
disabled	0.000	0.011	-0.197	-0.158	0.0000	-0.0018
student/military	-0.005	0.021	-0.342	-0.421	0.0018	-0.0087
part time	0.023	-0.001	-0.148	-0.152	-0.0034	0.0002
inactive	0.008	-0.005	-0.289	-0.171	-0.0022	0.0009
completed <3 years upper secondary	0.105	0.025	-0.076	-0.075	-0.0080	-0.0019
completed 3 years upper secondary	0.110	0.059	0.006	-0.022	0.0007	-0.0013
completed > 3 years upper secondary	0.089	0.078	0.206	0.211	0.0183	0.0165
missing data on education	-0.002	0.001	0.228	-0.220	-0.0004	-0.0002
region 2	-0.042	-0.025	-0.023	-0.013	0.0010	0.0003
region 3	-0.031	-0.017	-0.048	0.057	0.0015	-0.0009
region 4	-0.018	-0.002	-0.041	-0.048	0.0007	0.0001
region 5	-0.037	-0.028	-0.081	-0.088	0.0030	0.0024
region 6	-0.021	-0.038	-0.073	-0.075	0.0015	0.0028
population 2000-20000 individuals	-0.041	-0.022	0.036	0.045	**-0.0015**	-0.0010
population <2000 individuals	-0.104	-0.050	-0.086	-0.046	0.0089	0.0023
married	0.045	-0.034	0.095	0.158	0.0042	-0.0053
cohabiting	-0.003	-0.005	0.095	0.041	-0.0003	-0.0002
divorced	0.018	-0.008	-0.148	-0.203	-0.0027	0.0015
widow/widower	0.006	-0.007	-0.334	-0.191	-0.0021	0.0014
born_europe	0.000	-0.006	-0.015	-0.058	0.0000	0.0003
born_other	-0.007	0.006	-0.306	-0.239	0.0022	-0.0015
income	2.135	0.562	0.020	0.023	0.0427	0.0130
residual					0.0074	0.0288
CI, unstandardised					0.0543	0.0300

a) Elasticities are based on marginal effects from probit estimations, see Table 4.

b) Contributions that are statistically significant at 5%-level are in bold. Bootstrapped standard errors.

**Table 4:** Probability of private specialist visit partial effects after probit

	2000		2005	
	dF/dx	P >|z|	dF/dx	P >|z|
self-assessed health good	**0.0419**	0.006	**0.0665**	0.000
self-assessed health fair	**0.1053**	0.000	**0.1859**	0.000
self-assessed health poor or very poor	**0.2501**	0.000	**0.2556**	0.000
male, age 30-44	-0.0172	0.547	-0.0055	0.846
male, age 45-59	0.0394	0.224	-0.0063	0.834
male, age 60-69	**0.1202**	0.006	0.0334	0.385
female, age 16-29	**0.0876**	0.003	0.0149	0.586
female, age 30-44	**0.0954**	0.002	0.0324	0.270
female, age 45-59	**0.0913**	0.007	**0.1029**	0.002
female, age 60-69	0.0687	0.122	**0.0968**	0.022
disabled*	-0.0003	0.990	0.0200	0.457
student, military*	-0.0125	0.662	0.0359	0.214
Part time*	0.0244	0.202	-0.0014	0.947
inactive*	0.0124	0.606	-0.0072	0.769
completed <3 years upper secondary	**0.0528**	0.016	0.0143	0.543
completed 3 years upper secondary	**0.0679**	0.005	0.0311	0.211
completed > 3 years upper secondary	**0.0552**	0.023	0.0454	0.080
missing data on education	-0.0670	0.360	0.0037	0.925
region2*	-0.0262	0.143	-0.0141	0.444
region3*	-0.0364	0.072	-0.0201	0.355
region4*	-0.0165	0.406	-0.0014	0.944
region5*	**-0.0629**	0.006	**-0.0487**	0.048
region6*	-0.0332	0.149	**-0.0570**	0.013
population 2000-20000 individuals	-0.0267	0.079	-0.0138	0.392
population <2000 individuals	**-0.0543**	0.000	-0.0269	0.097
married*	0.0141	0.484	-0.0114	0.579
cohabiting*	-0.0036	0.875	-0.0040	0.847
divorced*	0.0440	0.165	-0.0172	0.555
widow_er*	0.0466	0.352	-0.0512	0.234
born_europe*	0.0024	0.943	-0.0195	0.525
born_other*	-0.0482	0.206	0.0272	0.455
Income	0.0288	0.073	0.0071	0.527
observed probability	0.164		0.157	
predicted probability	0.151		0.146	
N	3371		3002	
Pseudo-R2	0.053		0.051	
Log likelihood	-1427.1		-1238.4	

Contributions that are statistically significant at 5%-level are in bold. Bootstrapped standard errors.

We learn that the most important explanatory variables that contribute to pro-rich inequality are, in addition to income itself, dummies for having higher education and being a woman aged 45-59. Individuals in these groups have a high propensity of seeing a private specialist and are well represented among the better off; i.e., the concentration index of the variable is positive. Individuals with poor or very poor health also have a high positive elasticity, cet. par., but these individuals tend to belong to the poorer income groups. Therefore, they contribute negatively to the concentration index. There are clear geographical differences in the use of private specialists. Living outside the capital area is associated with lower use, especially for those who live in scarcely populated areas. However, concentration indices are so small that the impact on income-related inequality in use is limited. Covariates reflecting activity status do not seem important in explaining inequality because of low elasticities. The residual, i.e., the part of the concentration index that cannot be traced back to covariates included in the decomposition analysis, is large in 2005 both in absolute terms and especially relative to the total CI index. It has a positive sign, which implies that a lot of the positive association between income rank and use of private specialists is unexplained in 2005.

We have done an Oaxaca-type decomposition of change in CI between 2000 and 2005; see equations (5) and (6). The results are reported in Table 5. The total change to be explained is a decrease in inequality of 0.0243. Some of the variables that contribute significantly to the year-by-year indices are also important for explaining change. By far, the largest negative contribution to change comes from income because the elasticity of use with respect to income has been drastically reduced (the marginal effect of log of income is reduced from 2.9% to 0.7%, see Table 4). Dummies for self-assessed health also contribute to the decrease in inequality, but through another channel as their concentration indices have become more negative, meaning that individuals with worse than "very good health" have fallen behind in the income distribution. For instance, the concentration index for reporting poor or very poor health is -0.206 in 2000 versus -0.232 in 2005, and the decrease is even more drastic for the group reporting good health. The impact of a given change in the concentration index of a covariate depends on the level of the elasticity, which is large for all self-assessed health categories included. Living in a scarcely populated area has a smaller impact on pro-rich inequality in 2005 than in 2000. It is associated with low use of private specialist services and with low income, and thus contributes to pro-rich inequality in both years but to a lesser degree in 2005. Whether this is the result of a change in the concentration index or the elasticity depends on the base year used in the decomposition. The other variables reflecting geography, the regional dummies, show small changes each, but in sum, they contribute to a decrease in inequality. Large changes exist in contributions from the dummy for being married or from the combined dummy for being a student or doing military service. In both cases, changes are clearly the result of changes in the elasticities, but the forces behind them are different. Being a student or doing military service has a higher marginal effect in 2005 as well as forming a higher proportion of the 2005 sample, and these individuals are predominantly found within the low-income groups, thus their positive change in elasticity contributes to a decrease in inequality. For married people, the marginal effect declined as well as their sample proportion, and since they are well represented among the high-income groups, this caused income-related inequality to decrease. For both students/military service and married people, the marginal effects changed sign from 2000 to 2005, but in opposite directions; see Table 4. The change in sign is not important, although it is the direction of the change that matters.

**Table 5:** Change in inequality 2000-2005 in the probability of a private specialist visit, Oaxaca-type decomposition ^a)^

	Equation (5)		Equation (6)		
	changeCI*el	change_el*CI	changeCI*el	change_el*CI	total change CI
self-assessed health good	-0.0059	0.0007	-0.0041	-0.0012	-0.0052
self-assessed health fair	-0.0017	-0.0017	-0.0012	-0.0022	-0.0034
self-assessed health poor or very poor	-0.0022	-0.0005	-0.0021	-0.0006	-0.0027
male, age 30-44	0.0000	0.0003	0.0001	0.0003	0.0003
male, age 45-59	0.0002	-0.0094	-0.0014	-0.0078	-0.0092
male, age 60-69	0.0018	-0.0003	0.0052	-0.0036	0.0016
female, age 16-29	-0.0011	0.0096	-0.0060	0.0145	0.0085
female, age 30-44	0.0004	0.0037	0.0011	0.0030	0.0041
female, age 45-59	-0.0015	0.0017	-0.0014	0.0015	0.0002
female, age 60-69	0.0112	-0.0043	0.0067	0.0002	0.0069
disabled	0.0004	-0.0022	0.0000	-0.0018	-0.0018
student or doing military service	-0.0016	-0.0088	0.0004	-0.0108	-0.0104
part-time	0.0000	0.0035	-0.0001	0.0036	0.0035
inactive	-0.0006	0.0037	0.0009	0.0022	0.0031
completed <3 years upper secondary	0.0000	0.0061	0.0001	0.0060	0.0061
completed 3 years upper secondary	-0.0017	-0.0003	-0.0031	0.0011	-0.0020
completed > 3 years upper secondary	0.0004	-0.0022	0.0005	-0.0023	-0.0018
missing data on education	-0.0005	0.0007	0.0009	-0.0007	0.0002
region 2	-0.0003	-0.0004	-0.0004	-0.0002	-0.0007
region 3	-0.0017	-0.0007	-0.0033	0.0009	-0.0025
region 4	0.0000	-0.0007	0.0001	-0.0008	-0.0006
region 5	0.0002	-0.0008	0.0003	-0.0008	-0.0006
region 6	0.0001	0.0012	0.0000	0.0012	0.0013
population 2000-20000 individuals	-0.0002	0.0007	-0.0003	0.0009	0.0005
population <2000 individuals	-0.0020	-0.0046	-0.0041	-0.0025	-0.0066
married	-0.0021	-0.0074	0.0029	-0.0124	-0.0096
cohabitating	0.0003	-0.0002	0.0002	-0.0001	0.0001
divorced	0.0004	0.0038	-0.0010	0.0052	0.0042
widow/widower	-0.0011	0.0046	0.0009	0.0026	0.0036
born_europe	0.0003	0.0001	0.0000	0.0004	0.0004
b_other	0.0004	-0.0041	-0.0005	-0.0032	-0.0037
Income	0.0018	-0.0315	0.0067	-0.0364	-0.0297
residual					0.0214
% of change	25%	164%	8%	180%	
total change in CI					-0.0243

a) The table shows contributions to change in CI index, which are attributed to changes in the elasticity or concentration index of each covariate.

The decomposition analysis leaves the definition of "need" and inequity to the reader. But if we return to our original definition, which classifies all inequality that is not associated with health, age or gender as inequitable, cf. table 2 we learn from table 3 that the larger contributions to the decrease in inequity come from income and covariates reflecting geographical variation, as well as dummies for student/military and marital status.

## 7. Discussion and conclusion

Our finding that there is inequity in the use of specialist services is well in line with the results from the OECD research group [1]. In this study, we have refined the outcome variables, i.e., split specialist services into private specialist and hospital outpatient services, and arrived at the same results as Iversen and Kopperud did in their analysis using data from 2000 [3]. Clearly, inequity is found within the usage of private specialists. The current analysis extends the time frame of previous studies and benefits from more health information in the years 2002 and 2005. It shows that although inequity decreased substantially, it still existed in 2005.

The two studies mentioned above utilized self-assessed health, age and gender as the only health need indicators. There has been a discussion in the literature regarding how well the self-assessed health measure captures "need"; i.e., whether there is a reporting bias by socio-economic status so that inequity is systematically under-reported (for references, see [28,29]). Our results show that including a richer set of health status information with more detailed and perhaps "objective" measures makes the estimated inequality indices more pro-rich, which is consistent with such a reporting bias. We see it as an illustration of the argument that including more health information removes some omitted variable bias, e.g., unobserved heterogeneities that are correlated with both income and the use of health services [30]. Since our health delivery outcomes call for non-linear models, we have applied binary choice or count data models. Overall, the results are not sensitive to choice of estimator, ordinary least squares or non-linear, which is a general finding in the literature [22]. The estimated indices will depend on at what values of the covariates the marginal effects are estimated; see equation (2). To test the sensitivity of our results, we have estimated the HI indices at median values of the x's. For the probability of a private specialist visit, the indices are very similar whether estimated at mean or median values.

The bulk of the decrease in inequality is the result of changes in the elasticities of the covariates; that is, the driving force is not changes in how income is distributed with respect to the same covariates. Above all, the large decrease in the impact of income itself on the probability of using a private specialist is remarkable. It cannot be explained by changes in the real value of co-payments. This finding suggests that income is related to accessibility and use in a way that the included variables (for instance, age, education, geography) do not fully pick up. In general, we interpret the change in elasticities as an indication that access to private specialists is less rationed in 2005, as individuals with a low propensity of seeing a private specialist in 2000 increased their utilization. This holds for low-income individuals in general and specifically for some low-income groups identified in our data, like students or individuals living in scarcely populated areas.

One could argue that the general increase in activity levels observed among private specialist both within and outside hospitals improved access for everyone, but may have had the largest impact on groups that traditionally have had low use of private services. However, in our data, which are restricted to individuals aged below 70, we see no increase in the probability of a private specialist visit. Therefore a change in inequity cannot be driven by a change in activity levels alone. It seems reasonable to relate these findings to the reform in general practice, as having a regular GP may have made it easier to get a referral to a private specialist. The RGP scheme leads to increased capacity and improved access as well as increased stability in the doctor-patient relationship [31]. GPs themselves report that their role as gatekeepers has weakened because of the reform [17]. A priori, it is not obvious how a weaker gatekeeper role would affect the degree of inequity. One could expect this to favour individuals with high levels of education or high income who are often believed to be better at communicating their health problems with the GP [20]. However, there is no indication of such a development in our results. Therefore, it seems reasonable to point to factors that may have pulled in the opposite direction. Both improved access to a GP where discontinuity in the patient - doctor relationship was particularly frequent before the reform, and increased responsibility made explicit by contracted patient lists may have favoured low SES individuals the most.

Although regions have become more equal with respect to the use of private specialists, the impact of regional variation is small given that income is relatively equally distributed across the country. Thus, the potential for the hospital ownership reform to have had any impact on income-related inequality is limited. Furthermore, no large changes in the allocation of resources by region occurred after the 2002 reform. However, state ownership of hospitals may have had an indirect effect on equity [32]. suggests that the state's taking over of financial responsibility may have contributed to a rise in activity through supplementary funding of RHSs, and that the impact on equity from the hospital reform is inconclusive.

Given that removing inequity is a declared ambition in health policy, the development unravelled in this analysis should be welcomed. Specialist services are more equally distributed in 2005 than in 2000, the inequity in use of private specialists is less and there is an increase in mean use of hospital outpatient visits, which is equitably distributed. There is still significant inequity in the distribution of private specialist visits as well-off individuals have a higher propensity to be treated by a specialist than less well-off individuals of equal need. The most important factor behind the decrease in horizontal inequity from 2000 to 2005 is the decrease in the marginal effect of income on utilization. Still, income and having higher education are the two covariates that give the major contributions to the observed pro-rich inequality in 2005.

In essence, this analysis indicates that organisational reforms can reduce inequity in health care utilization, but it also reveals that there are still challenges to be met in this respect.

## Competing interests

The authors declare that they have no competing interests.

## Authors' contributions

The authors contributed equally to this work
